# The Effect of Activated FXIII, a Transglutaminase, on Vascular Smooth Muscle Cells

**DOI:** 10.3390/ijms23105845

**Published:** 2022-05-23

**Authors:** Réka Bogáti, Éva Katona, Amir H. Shemirani, Enikő Balogh, Helga Bárdos, Viktória Jeney, László Muszbek

**Affiliations:** 1 Division of Clinical Laboratory Science, Department of Laboratory Medicine, Faculty of Medicine, University of Debrecen, 98 Nagyerdei Krt, H-4032 Debrecen, Hungary; bogati.reka@med.unideb.hu (R.B.); ekatona@med.unideb.hu (É.K.); shemirani1@gmail.com (A.H.S.); 2 Research Center for Molecular Medicine, Faculty of Medicine, University of Debrecen, 98 Nagyerdei Krt, H-4032 Debrecen, Hungary; balogh.eniko@med.unideb.hu (E.B.); jeney.viktoria@med.unideb.hu (V.J.); 3 Department of Public Health and Epidemiology, Faculty of Medicine, University of Debrecen, Egyetem Tér 1, H-4032 Debrecen, Hungary; bardos.helga@med.unideb.hu

**Keywords:** factor XIII, vascular smooth muscle cell, cell proliferation, collagen secretion, thrombospondin-1

## Abstract

Plasma factor XIII (pFXIII) is a heterotetramer of FXIII-A and FXIII-B subunits. The cellular form (cFXIII), a dimer of FXIII-A, is present in a number of cell types. Activated FXIII (FXIIIa), a transglutaminase, plays an important role in clot stabilization, wound healing, angiogenesis and maintenance of pregnancy. It has a direct effect on vascular endothelial cells and fibroblasts, which have been implicated in the development of atherosclerotic plaques. Our aim was to explore the effect of FXIIIa on human aortic smooth muscle cells (HAoSMCs), another major cell type in the atherosclerotic plaque. Osteoblastic transformation induced by Pi and Ca^2+^ failed to elicit the expression of cFXIII in HAoSMCs. EZ4U, CCK-8 and CytoSelect Wound Healing assays were used to investigate cell proliferation and migration. The Sircol Collagen Assay Kit was used to monitor collagen secretion. Thrombospondin-1 (TSP-1) levels were measured by ELISA. Cell-associated TSP-1 was detected by the immunofluorescence technique. The TSP-1 mRNA level was estimated by RT-qPCR. Activated recombinant cFXIII (rFXIIIa) increased cell proliferation and collagen secretion. In parallel, a 67% decrease in TSP-1 concentration in the medium and a 2.5-fold increase in cells were observed. TSP-1 mRNA did not change significantly. These effects of FXIIIa might contribute to the pathogenesis of atherosclerotic plaques.

## 1. Introduction

Coagulation factor XIII (FXIII), a protransglutaminase, exists in two forms, one of which is a circulating plasma protein (pFXIII), while the other is an intracellular cytoplasmic constituent expressed in several cell types (cFXIII) [1,2,3]. pFXIII is an inactive heterotetramer that consists of two potentially active A subunits (FXIII-A) and two inhibitory/protective B subunits (FXIII-B). The heterotetramer (FXIII-A_2_B_2_) is transformed into an active transglutaminase (FXIIIa) (protein-glutamine:amine γ glutamyltransferase, EC2.3.2.13; TG) by the concerted action of thrombin and Ca^2+^. FXIIIa catalyzes an acyl transfer reaction. In this reaction, the carboxamide group of a peptide-bound glutamine residue is the acyl donor, and a primary amine is the acyl acceptor. If the primary amine happens to be an ε amino group of a peptide-bound lysine residue, the end result is the covalent cross-linking of two peptide chains through an ε(γ glutamyl)lysyl isopeptide bond (for further details, see reviews [4,5]).

cFXIII is a homodimer of FXIII-A (FXIII-A_2_) with cytoplasmic localization [1], which has been identified in platelets [6,7,8], monocytes/macrophages [9,10], osteoblasts, osteoclasts, osteocytes [11,12,13], chondrocytes [14,15], preadipocytes [16] and corneal keratocytes [17]. It does not require the proteolytic removal of the activation peptide for activation; the elevation of intracellular Ca^2+^ concentration is sufficient to bring about the active configuration [18,19,20].

The most well-studied function of FXIIIa is the cross-linking of fibrin chains and the covalent attachment of α_2_-plasmin inhibitor to fibrin. These functions are essential for maintaining hemostasis by making mechanically stable fibrin clots that are resistant to degradation by the fibrinolytic machinery. Due to the lack of these functions, FXIII-A-deficient patients suffer from severe bleeding diathesis. FXIIIa has diverse functions that involve several direct or indirect cellular effects (see details in recent reviews [1,21]). FXIII-deficient women fail to carry out pregnancy [22,23]. The first reported FXIII-deficient patient demonstrated impaired wound healing [24], and in subsequent studies, 15–30% of such patients showed a similar abnormality. The prolonged healing of excisional wounds and impaired tissue repair in FXIII-A knockout mice confirmed the importance of FXIII in the wound healing process [25].

In a series of publications from Inbal’s laboratory, it was demonstrated that FXIIIa exerts a proangiogenic effect that is associated with the suppression of thrombospondin-1 (TSP-1) and involves the stimulation of endothelial cell proliferation, migration and inhibition of apoptosis [26,27]. TSPs are multifunctional matricellular glycoproteins with complex multi-domain structures that permit interaction with different individual partners [28,29]. The TSP family comprises five members (TSP-1-5) with similar C-terminal structural elements. Based on different N-termini, they are divided into three different categories: TSP-1 and TSP-2 have trimeric structures (subgroup A), while the rest of the TSPs are pentameric. Among TSPs, the structure, interactions and functions of TSP-1 are the most well explored and characterized. TSP-1 is expressed in a number of cell types in response to injury or in the process of remodeling. Its effect depends on the availability of its numerous interacting partners, including surface receptors and extracellular matrix proteins. Among others, it binds to integrins, transforming growth factor-β (TGF-β), CD36, CD47, Ca^2+^ and heparin. Its significance in regulating the process of angiogenesis as an antiangiogenic factor has been well established [30,31]. TSP-1 is a substrate of FXIIIa that can incorporate low-molecular-weight amines into the protein and can induce the formation of covalent homo-polymers [32].

Monocytes and fibroblasts have an important role in the formation of atherosclerotic plaques. FXIIIa enhanced migration and proliferation and inhibited apoptosis of monocytes and fibroblasts [33]. The relation of FXIII to the other major cellular component of the plaque, vascular smooth muscle cells (VSMCs), has been only partially explored. Depending on environmental factors, VMSCs in the atherosclerotic plaque might undergo phenotypic switching. VSMCs in the media originally represent the contractile phenotype. These cells then migrate, and in the vascular microenvironment, they become transitional de-differentiated VSMCs. The transitional cells then can assume the characteristics of several cell types, including osteoblasts, chondrocytes, adipocytes, foam cells and senescent cells [34,35,36,37].

In the present study, we addressed the following questions:

1. Does the osteoblastic transformation of human aortic smooth muscle cells (HAoSMC) result in the expression of cFXIII?
2. How does FXIIIa influence the proliferation, migration and collagen secretion of HAoSMCs?
3. Does FXIIIa influence intracellular and cell-associated TSP-1 content of HAoSMCs and their TSP-1 synthesis?

## 2. Results

### 2.1. Osteoblastic Transformation Does Not Induce FXIII-A Expression in HAoSMCs

**Figure 1:**
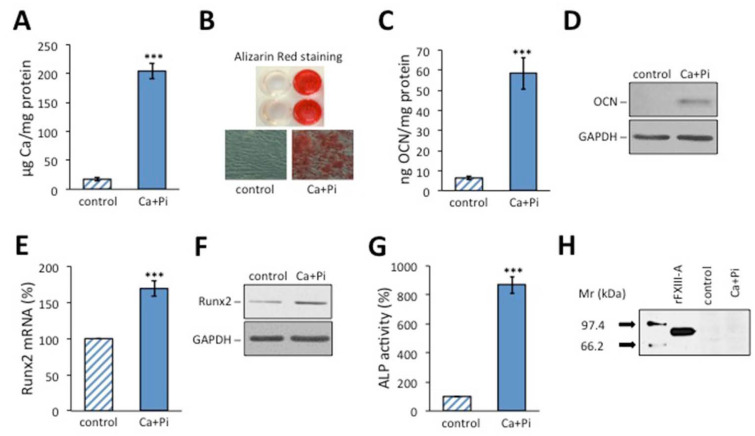
The lack of cellular FXIII (cFXIII) expression in human aortic smooth muscle cells undergoing osteoblastic transformation. Osteoblastic transformation was induced by 2.5 mM inorganic phosphate (Pi) and 1.2 mM Ca^2+^ in the culture medium, and successful transformation was tested with various osteoblastic markers: Ca deposition was assessed by Ca assay (**A**) and Alizarin Red staining (**B**). Osteocalcin incorporation into the extracellular matrix was measured by ELISA (**C**) and visualized by Western blot (**D**). Runx2 mRNA (**E**) and protein (**F**) expression levels were detected by quantitative reverse polymerase chain reaction and by Western blotting, respectively. The expression of the latter two parameters was normalized to glyceraldehyde 3-phosphate dehydrogenase (GAPDH). Alkaline phosphatase activity was measured by enzymatic assay (**G**). The lack of FXIII expression in cells undergoing osteoblastic transformation is revealed by Western blotting (**H**). Intense staining of rFXIII-A standard by Western blotting is clearly shown in the figure. *** *p* < 0.001.

As FXIII-A is a characteristic component of human osteoblasts [11,12,38], first, we investigated whether osteoblastic transformation of HAoSMCs is accompanied by the induction of FXIII-A synthesis. Osteoblastic differentiation of the cells induced by a calcification medium (CM) containing 2.5 mM Pi and 1.2 mM Ca^2+^ is demonstrated in Figure 1A–G using five different osteoblastic markers. The accumulation of Ca deposits, as shown by Alizarin Red S staining (Figure 1A,B), and the increased synthesis of osteocalcin (Figure 1C,D) and Runx2 (Figure 1E,F), together with the robust elevation of alkaline phosphatase (ALP) activity (Figure 1G), clearly show that the cells have undergone osteoblastic differentiation. However, no FXIII-A could be detected in the cell lysate by Western blotting (Figure 1H) or by FXIII-A ELISA (not shown).

### 2.2. rFXIIIa Enhances the Proliferation of HAoSMCs

**Figure 2:**
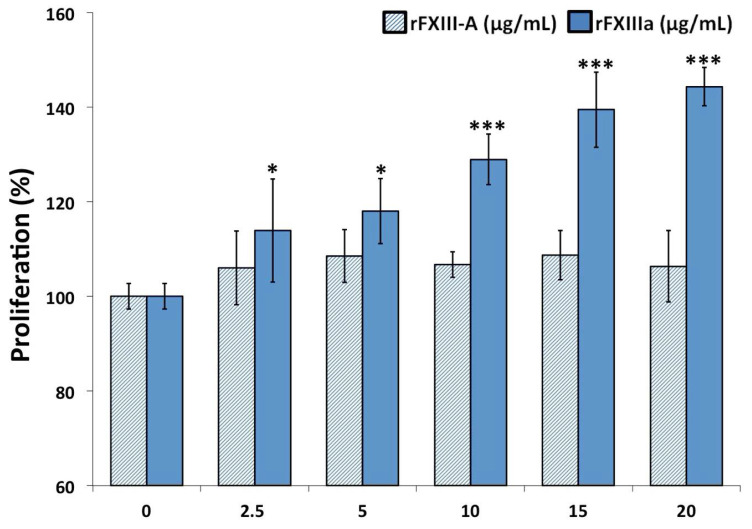
rFXIIIa enhances the proliferation of human aortic smooth muscle cells (HAoSMCs). The proliferation of rFXIIIa-treated cells is expressed as percentage of the proliferation of non-treated cells. As demonstrated by shaded columns, non-activated rFXIII-A_2_ failed to influence the proliferation of HAoSMCs. Cell proliferation was quantified by EZ4U assay based on measuring the reduction of tetrazolium salt to colored formazan at 450 nm. The statistical comparison is between time 0 and the respective time points; * *p* < 0.05, *** *p* < 0.001.

In the rest of the experiments, the effect of rFXIIIa externally administered to HAoSMCs was investigated. The extracellular concentration of FXIIIa in a hemorrhagic plaque is not known, but one may suspect a concentration in the range from below to somewhat above its plasma concentration (10 μg/mL); therefore, we used the concentration range 2.5–20 μg/mL in our experiments. Non-activated recombinant FXIII-A_2_, even at double plasma concentration, failed to influence the proliferation of HAoSMCs (Figure 2), while rFXIIIa, even below the mean plasma FXIII-A concentration, significantly enhanced cell proliferation, as revealed by the EZ4U assay. Practically the same results were obtained with the CCK-8 cell proliferation assay (Supplementary Figure). In these experiments, rFXIII-A_2_ was activated by thrombin and Ca^2+^. As thrombin itself can enhance cell proliferation, in the experiments, following a 10 min FXIII activation period, thrombin was blocked by lepirudin, a recombinant hirudin derivative. In none of the experiments described below did lepirudin-blocked thrombin exert any effect on HAoSMCs.

### 2.3. rFXIIIa Accelerates the Closure of In Vitro Gap Wound

**Figure 3:**
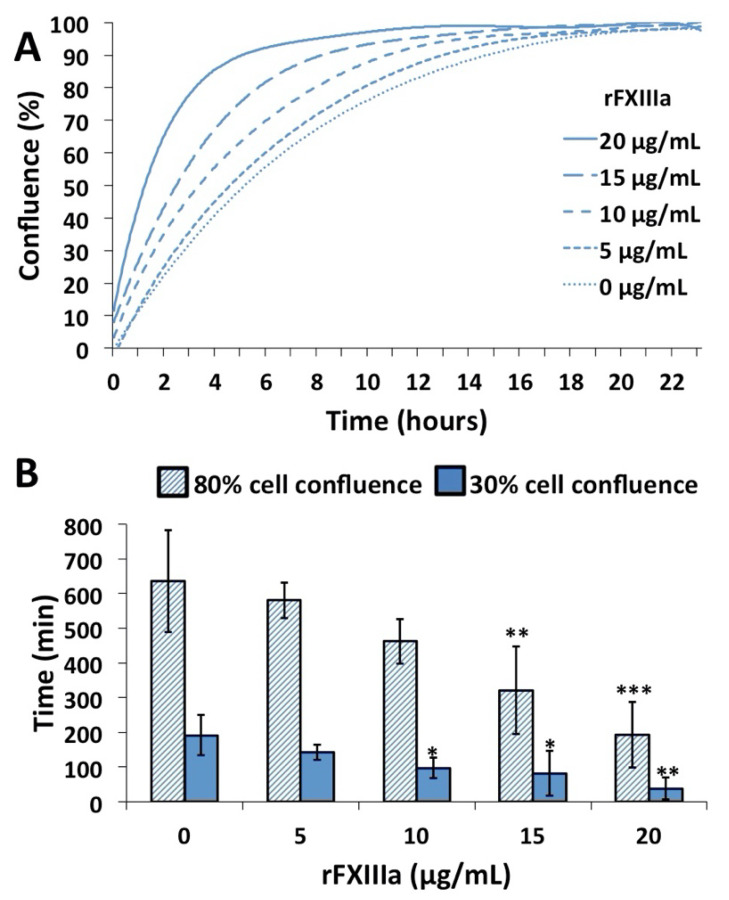
rFXIIIa accelerates the closure of in vitro gap wound in human aortic smooth muscle cell culture. (**A**) After removing the insert from the cell culture, the gradual closure of the cell-free gap by proliferating and migrating cells at various rFXIIIa concentrations was continuously monitored by real-time microscopy. (**B**) The time required for reaching 30 and 80% cell confluence was determined. The statistical comparison is between time 0 and the respective time points; * *p* < 0.05, ** *p* < 0.01, *** *p* < 0.001.

Next, we tested the effect of rFXIIIa in an in vitro wound closure assay. In this experimental set-up, proliferating and migrating cells gradually fill up the originally empty (cell-free) area of the removed insert, and the kinetics of the gap closure can be monitored (Figure 3A,B and Supplementary Video). The rate at which the gap was covered was highly accelerated by the addition of rFXIIIa (Figure 3A and Supplementary Video), and there was a rFXIIIa concentration-dependent shortening of the time required to reach 30% or 80% cell confluence (Figure 3B).

### 2.4. rFXIIIa Induced Elevated Collagen Secretion by HAoSMCs

**Figure 4:**
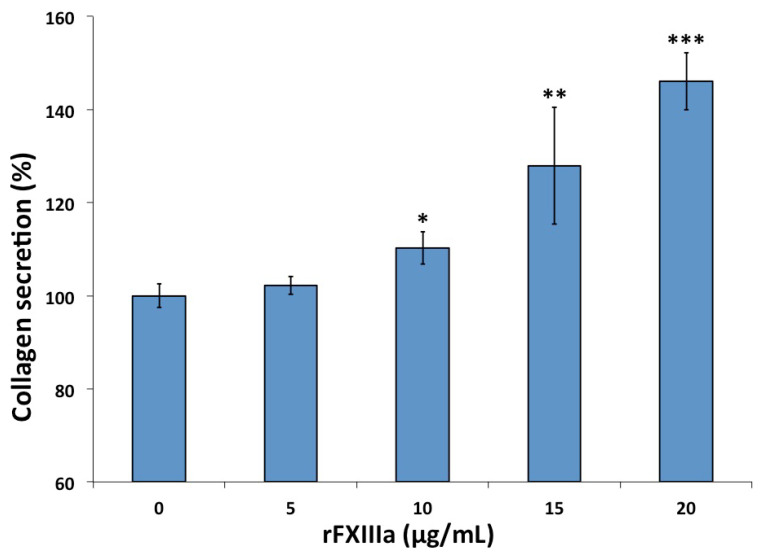
Collagen secretion of human aortic smooth muscle cells is enhanced by rFXIIIa. Non-activated rFXIII-A_2_ was without effect (not shown on the figure); * *p* < 0.05, ** *p* < 0.01, *** *p* < 0.001.

Twenty-four hours following three daily treatments of HAoSMCs with various concentrations of rFXIIIa, their secretion of collagen, a key component of the extracellular matrix, became elevated (Figure 4). As opposed to the cell proliferation experiments, here, higher rFXIIIa concentrations (10 μg/mL or above) and repeated treatments were required to reach statistically significant changes.

### 2.5. TSP-1 in the Culture Medium and Cell-Associated TSP-1 Following rFXIIIa Treatment of HAoSMCs

**Figure 5:**
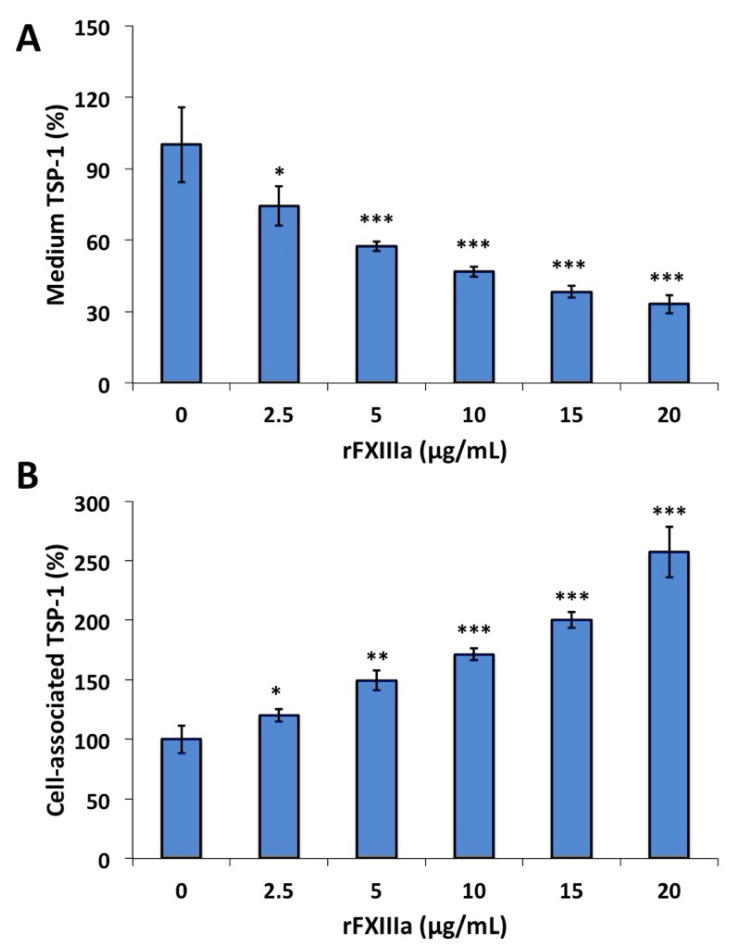
The influence of rFXIIIa on the distribution of thrombospondin-1 (TSP-1) between the culture medium and the cells. (**A**) Treatment with rFXIIIa decreases TSP-1 concentration in the medium. (**B**) rFXIIIa induces the elevation of cell-associated TSP-1 content. Cell-associated TSP-1 includes TSP-1 in the intracellular compartment and TSP-1 bound to the cells and cannot be removed together with the medium or by repeated washings of the cultured cells. In both cases, TSP-1 measured in the absence of rFXIIIa was taken as 100%, and values obtained following treatment with rFXIIIa were calculated accordingly; * *p* < 0.05, ** *p* < 0.01, *** *p* < 0.001.

Twenty-four hours after a single treatment, even the lowest rFXIIIa concentration (2.5 μg/mL) resulted in a significant decrease in TSP-1 in the culture medium (Figure 5A). Having treated the cells with relatively high concentrations of rFXIIIa (15–20 μg/mL), the amount of TSP-1 in the medium decreased to one-third of that measured in the medium of non-treated cells. As opposed to the medium, changes in the opposite direction were observed in the amount of TSP-1 in HAoMSC lysates. In this case, the TSP-1 content of the cells gradually increased in parallel with increasing rFXIIIa concentration in the culture medium (Figure 5B).

The elevation of cellular TSP-1 might be due to increased de novo synthesis of the protein or to the association of TSP-1 originally present in the medium with the cells. First, we calculated the total TSP-1 content per well of the tissue culture plates in which cells were cultured with or without rFXIIIa. The total amount of TSP-1 in a well includes TSP-1 in the medium, intracellular TSP-1 and TSP-1 that remains associated with the cells after the removal of the medium. rFXIIIa, even at the highest concentration, induced only a slight non-significant decrease in the total TSP-1 as compared to the total TSP-1 in wells containing non-treated HAoSMC (135.4 ± 11.4 ng/well versus 153.1 ± 13.1 ng/well; n = 3, *p* > 0.05).

**Figure 6:**
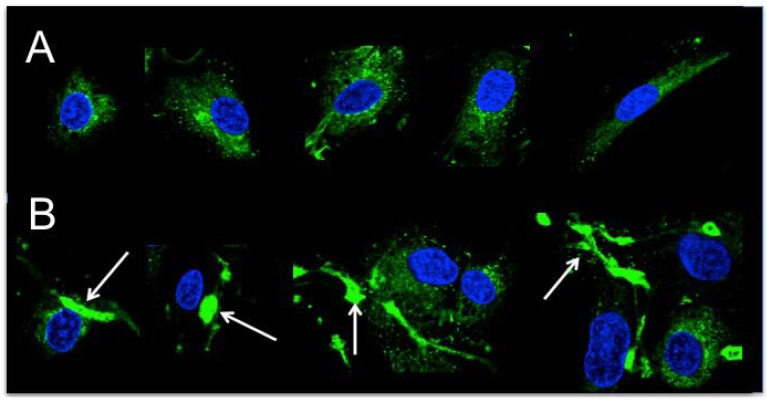
Demonstration of thrombospondin-1 (TSP-1) in human aortic smooth muscle cells (HAoSMCs). (**A**) Immunofluorescent visualization of TSP-1 in non-treated cells; (**B**) intracellular and cell-associated TSP-1 in HAoSMCs treated with 20 μg/mL rFXIIIa. White arrows indicate granule-like formations intensively stained for TSP-1. Most frequently, they were closely attached to cells, but occasionally they also existed independently.

Figure 6A demonstrates the even cytoplasmic distribution of TSP-1 in non-treated individual cells. In contrast, in the presence of rFXIIIa, numerous granules intensively stained for TSP-1 were observed, most of which were associated with cells (Figure 6B).

**Figure 7:**
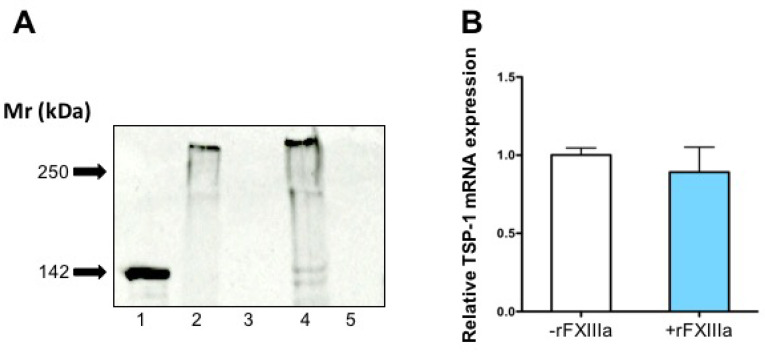
The effect of rFXIIIa on cellular and cell-associated TSP-1 and on TSP-1 mRNA in HAoSMC. (**A**) The increase in intracellular plus cell-associated TSP-1 in HAoSMCs following rFXIIIa treatment was demonstrated by reducing SDS-PAGE and Western blotting technique. Lane 1: 200 ng TSP-1 standard; lane 2: lysate of GM-treated cells (54 μg protein); lane 3: extracellular matrix of GM-treated cells (2 μg protein); lane 4: lysate of cells treated with 20 μg/mL rFXIIIa (54 μg protein); lane 5: extracellular matrix of rFXIIIa-treated cells (2 μg protein). The great majority of disulfide-linked TSP-1 monomers stay together as high-molecular-weight homotrimers. TSP-1 monomers are represented only by faint bands with a mobility of around 150 kD. (**B**) TSP-1 mRNA content of rFXIIIa-treated cells was compared to the TSP-1 mRNA content of GM-treated control cells.

Cellular and cell-associated TSP-1 was predominantly represented by high-molecular-weight bands on the Western blots (Figure 7A). Densitometric analysis of the Western blot revealed a 2.5-fold increase in cell-associated TSP-1 with FXIIIa treatment. No TSP-1 showed up in the Western blot of the extracellular matrix. These findings suggest that treatment with rFXIIIa results in the increased association of TSP-1 with the cells rather than inducing de novo TSP-1 synthesis. This statement is supported by the lack of significant change in the intracellular concentration of TSP-1 mRNA (Figure 7B).

## 3. Discussion

In addition to their normal contractile phenotype present in the medial layer of the vessel wall, VSMCs may adopt several alternative phenotypes exerting various functions in physiological and/or pathological conditions (reviewed in references [34,35,36,37,39]). The synthetic phenotype is important for tissue repair by promoting cell proliferation and collagen synthesis. The osteogenic/chondrogenic phenotype is involved in calcification. VSMC-derived foam cells represent a macrophage-like phenotype, while the senescent phenotype is the product of inflammatory processes. As cFXIII is a component of osteoblasts, first, we addressed the question of whether osteoblastic transformation of VSMCs is accompanied by the expression of FXIII-A. However, VSMCs undergoing osteoblastic transformation, as demonstrated by the expression of genetic and proteomic osteoblast markers, failed to express cFXIII.

As VSMCs themselves do not produce cFXIII, in further experiments, we tested whether their function is influenced by external FXIII. Extracellular FXIII inside the vessel wall might be derived from plasma leaving the bloodstream as the consequence of plaque hemorrhage. cFXIII derived from blood platelets [40] and plaque macrophages [41] might also represent a source of cFXIII within the plaque. Finally, in activated platelets and macrophages, cFXIII might be translocated from the cytoplasm to the cell surface [1,42,43,44]. Thrombin generated within the plaque [45] can activate FXIII, and cFXIII might be transformed into its active form in the intracellular compartment of activated platelets and macrophages by the non-proteolytic mechanism [2]. In situ–formed or cell-derived FXIIIa might exert its effect on VSMCs. In the rest of this study, the effect of activated FXIII on HAoSMCs was explored, testing several VSMC functions that are important from the point of view of the development and sustainment of atherosclerotic plaque. The assays included the measurement of cell proliferation, in vitro gap closure, collagen secretion and the distribution of TSP-1.

The results obtained with two kits measuring cell proliferation demonstrated that FXIIIa, even well below the concentration of FXIII in the plasma, enhanced the proliferation of HAoSMCs. These results were further supported by the gap closure assay. In the latter assay, the combined effect of FXIIIa on the cell proliferation and migration of cells was investigated. A video demonstrating the kinetics of filling up the empty area that was left after removing the insert from the cell culture clearly suggests that in addition to promoting cell proliferation, FXIIIa also enhanced the motility of HAoSMCs. The active form of FXIII with transglutaminase activity was required for exerting these effects, and non-activated FXIII failed to influence the migration/proliferation of HAoSMCs. The cellular effect of FXIII was investigated in a few other cell types by Inbal’s group. They showed that at a somewhat higher concentration (50 µg/mL) than the one that we used in the present experiments, activated plasma FXIII increased the migration and proliferation and decreased the apoptosis of endothelial cells and monocytes [26,33]. Its effect on fibroblasts was less clear. The migration but not the proliferation of skin fibroblasts was considerably enhanced by FXIIIa, while its effect on lung fibroblasts, when compared to iodoacetamide-inhibited FXIIIa, was only marginal [33]. FXIIIa exerts its effect on endothelial cells, monocytes and skin fibroblasts by binding to and cross-linking the vitronectin receptor (α_v_β_3_ integrin). As VSMCs also possess the vitronectin receptor [46,47], it seems likely that the effect of FXIIIa on these cells follows a similar pattern. However, the exact mechanism is still to be confirmed. It is to be noted that FXIII used in the experiments was activated by thrombin, and thrombin itself might influence the investigated VSMCs’ functions. Comparison to FXIIIa blocked by iodoacetamide is one way to test the effect of FXIIIa transglutaminase. In this case, thrombin-cleaved and active-site-blocked FXIIIa is present in the control/blank incubation mixture. Instead, we chose to block thrombin activity following the FXIII activation period. This way, intact, non-activated FXIII remained in the control sample. No effect of intact cFXIII could be observed.

The effect of FXIIIa on collagen secretion has been previously investigated in osteoblasts. It was demonstrated that irreversible inhibition of FXIIIa transglutaminase but not transglutaminase 2 is highly potent in inhibiting osteoblast differentiation and mineralization and reduces the secretion of both fibronectin and type I collagen and their release from the cell surface [13]. In our experiments, collagen secretion and deposition into the extracellular matrix were monitored at various FXIIIa concentrations. A somewhat higher FXIIIa concentration was required to obtain a significant increase in collagen deposition than that which was required to increase the proliferation of VSMCs. FXIIIa might be involved in modulating the contractile phenotype of VSMCs toward the synthetic phenotype through a fate switch to transitional multi-potential cells, which then, via collagen matrix deposition, adopt a plaque-stabilizing character [35].

Several in vitro and in vivo studies demonstrated that FXIIIa in part exerts its effect on angiogenesis by influencing the TSP-1 level and TSP-1 synthesis [26,27,48,49]. The treatment of human vein endothelial cells with FXIIIa resulted in the disappearance of TSP-1 mRNA, a significant reduction in TSP-1 secretion and a moderate decrease in TSP-1 synthesis. In an in vivo model, FXIIIa-induced neovascularization of rabbit cornea was associated with the disappearance of TSP-1. In another in vivo angiogenesis model using heterotopic mouse heart allografts, treatment with FXIIIa significantly decreased TSP-1 mRNA in heart allografts [48].

The present study confirms that FXIIIa, in addition to its effect on endothelial cells, fibroblasts and macrophages, also influences VMSCs, which are important constituents of atherosclerotic plaque. It enhances the proliferation, migration and collagen secretion of HAoSMCs. Such effects are important for increasing plaque stability but may also increase the size of the plaque. The effect of FXIIIa on the TSP-1 of VSMCs is more complex. TSP-1 in the medium is significantly decreased, which may contribute to the proangiogenic effect of FXIIIa. However, such a decrease does not seem to be related to decreased synthesis, like in other cell types, but part of TSP-1 that is secreted remains associated with the cells. The role of cell-associated TSP-1 requires further elaboration.

## 4. Methods

### 4.1. Materials

Recombinant FXIII-A_2_ (rFXIII) was a kind gift from Novo Nordisk (Måløv, Denmark). Human thrombin was purchased from CoaChrom (Maria Enzersdorf, Austria). Fetal bovine serum (FBS) was obtained from Life Technologies (Waltham, MA, USA). A very low residual amount of FXIII-A was detectable in FBS by a highly sensitive ELISA method [50]. In the medium containing 5% FBS, the FXIII-A concentration was 19.75 ng/mL, which corresponded to 0.2% plasma FXIII-A concentration. For certain experiments, FBS was depleted even from that low amount of FXIII-A by immuno-absorption chromatography using an anti-FXIII-A 3B2H12 mouse monoclonal antibody [50] that cross-reacts with bovine FXIII-A. The antibody was covalently bound to CNBr-activated Sepharose 4B gel (GE Healthcare Bio-Sciences AB, Uppsala, Sweden), according to the protocol recommended by the manufacturer. After the depletion procedure, FXIII-A concentration of FBS was below the limit of detection.

### 4.2. Cell Culture

Human aortic smooth muscle cells (HAoSMCs) were obtained from Cell Applications (San Diego, CA, USA) and were maintained in high-glucose Dulbecco’s Modified Eagle Medium (Sigma Aldrich, St Louis, MO, USA) containing 1 mM sodium-pyruvate, 4 mM L-glutamine and 116 μg/mL gentamycin (growth medium: GM). GM supplemented with 5% FBS was replaced every other day. Cells were grown to confluence and used from passages 5 to 9.

### 4.3. Assessment of Osteoblastic Differentiation

Osteoblastic transformation of HAoSMCs seeded on six-well plates was induced by calcification medium (CM) containing 2.5 mM inorganic phosphate (Pi), 1.2 mM Ca^2+^ and 3% FBS in GM. Cells cultured in GM were used as controls. After 24 h, GM was replaced by CM. Two days later, the cells received a second CM treatment, 48 h after which the cells were tested for osteoblastic differentiation markers. Calcium deposition was measured by QuantiChrome Calcium Assay Kit [51] (Gentaur, Brussels, Belgium). Mineral deposition in the extracellular matrix was assessed by Alizarin Red S staining [3]. Osteocalcin content of the extracellular matrix was determined by Western blot [52] analysis and ELISA (Bender MedSystems, Burlingame, CA, USA) [53]. Runx2 mRNA and Runx2 protein expression levels were evaluated by quantitative reverse transcription-polymerase chain reaction and Western blot analysis, respectively [3]. Alkaline phosphatase activity in the cell lysates was measured using ALP Yellow Liquid Substrate (Sigma Aldrich, St Louis, MO, USA) [52]. Detection of cellular cFXIII in the cell lysates was measured by ELISA [50] and detected by Western blotting. In the latter case, lysates of the same protein content from non-treated HAoSMCs and HAoSMCs undergoing osteoblastic transformation were analyzed. Sheep anti-human FXIII-A antibody (Affinity Biologicals, Ancaster, Canada) and Vectastain ABC HRP kit containing biotinylated anti-sheep IgG and streptavidin–horseradish peroxidase complex (Burlingame, CA, USA) were used for FXIII-A labeling, and ECL Western Blotting Detection Reagent (Sigma Aldrich, St Louis, MO, USA) was used for visualization.

### 4.4. Treatment of Cells with Activated rFXIII

rFXIII-A_2_ (20 μg/mL) was activated in GM containing 0.2% FXIII-depleted FBS, 5 U/mL human thrombin and 1 mM CaCl_2_ for 10 min at 37 °C. After activation, thrombin was blocked by 50 ATU/mL lepirudin (Refludan; Pharmion, Windsor, UK), a recombinant hirudin derivative, for 10 min at 37 °C. Activated rFXIII-A_2_ (rFXIIIa) was further diluted (to 15, 10, 5 and 2.5 μg/mL) in GM containing 0.2% FXIII-depleted FBS, 1 mM CaCl_2_ and 5 U/mL thrombin inhibited by 50 ATU/mL lepirudin. Twenty-four hours after cell seeding in specific culture plates, GM was exchanged with media containing different amounts of rFXIIIa or non-activated rFXIII-A. In control experiments, only lepirudin-inhibited thrombin and CaCl_2_ were added to the cells. In certain experiments, treatments were repeated daily for three days.

### 4.5. Cell Proliferation Assays

HAoSMCs were seeded into 96-well plates at a density of 0.6 × 10^4^ cells/well. Twenty-four hours after seeding, cells were treated with rFXIIIa or non-activated rFXIII. Treatments were repeated daily for 3 days. The extent of proliferation was determined 24 h after the last treatment by using EZ4U Cell Proliferation and Cytotoxicity Assay (Biomedica, Vienna, Austria) and also by CCK-8 Cell Counting kit (Enzo Life Sciences, Farmingdale, NY, USA). Both kits are based on the reduction of tetrazolium salt to colored formazan. Absorbance was measured at 450 nm after a 4 h incubation at 37 °C.

### 4.6. In Vitro Wound Healing Assay

CytoSelect™ 24-Well Wound Healing Assay (Cell Biolabs, San Diego, CA, USA) was used for monitoring in vitro wound closure. Each well contained a specific insert, around which 20 × 10^4^ cells/well were seeded. The cultured cells form a monolayer within 24 h. When the insert is removed, the remaining wound gap becomes gradually covered by migrating and proliferating cells. In this experiment, the media contained 5% FBS to achieve proper binding of the cell monolayer and to prevent detachment during subsequent treatments. HAoSMC monolayers were treated with various concentrations of rFXIIIa. Inserts were removed after 24 h of treatment. Wound gap closure was recorded for 24 h by measuring cell confluence in Juli Stage Real Time Microscope (NanoEnTek, Seoul, South Korea). Three different positions in each well and three parallel wells were analyzed for each individual treatment.

### 4.7. Collagen Deposition in the Extracellular Matrix of HAoSMC

HAoSMCs were seeded into 12-well plates at a density of 10 × 10^4^ cells/well. After 24 h, they were treated with various concentrations of rFXIIIa daily for 3 days. Sircol Collagen Assay Kit (Biocolor, Carrickfergus, UK) was used for the measurement of collagen secreted into the extracellular matrix.

### 4.8. Measurement of Thrombospondin-1 in the Lysate and Culture Media of HAoSMCs by ELISA

HAoSMCs were cultured and treated as described for the cell proliferation assay. TSP-1 concentrations were measured by ELISA (ThermoFisher, Frederick, MD, USA) in the cell supernatants and in the lysates of washed cells 24 h after the treatment. Cell lysis buffer contained 1% Triton-X 100 and 10% of the solution prepared from SigmaFast Protease Inhibitor Tablet (Sigma Aldrich, St Louis, MO, USA). After centrifugation of cell lysates, supernatants were used for the measurement of TSP-1.

### 4.9. Detection of Thrombospondin-1 in HAoSMCs and in Their Extracellular Matrix by Western Blotting

HAoSMCs were seeded into a 6-well plate at a density of 50 × 10^4^ cells/well. Cells were treated with 20 μg/mL rFXIIIa or with GM for 24 h. After discarding the supernatants and washing the cells with PBS, cells were detached by adding PBS containing 2 mM EDTA and 10% protease inhibitory cocktail (Biotool, Munich, Germany) for 30 min at 37 °C. Detached cells were collected by centrifugation. Cell pellets were lysed in radio-immunoprecipitation assay (RIPA) buffer (0.1% SDS, 0.5% deoxycholate and 1% Triton X-100), and protein concentrations were determined by Pierce™ BCA protein assay (ThermoFisher, Waltham, MA, USA). The extracellular matrix remaining after cell detachment was washed with PBS containing 2 mM EDTA and 10% protease inhibitory cocktail and then dissolved by shaking in SDS-PAGE sample buffer for 30 min at room temperature. Samples were concentrated using Microcon-10 kDa Centrifugal Filter Unit (Millipore, Darmstadt, Germany). The protein concentrations of the concentrates were determined. Samples were analyzed by SDS-PAGE and Western blotting. Biotinylated mouse monoclonal anti-TSP-1 IgG (ThermoFisher), HRP-conjugated goat polyclonal anti-mouse IgG (Bio-Rad, Hercules, CA, USA) and ECL chemiluminescent reagent were used for detection.

### 4.10. Immunofluorescent Staining of HAoSMCs for Thrombospondin-1

Cells were seeded into Clipmax T 10 cm^2^ flasks (Techno Plastic Products, Trasadingen, Switzerland) at a density of 8 × 10^4^ cells/flask. Cells were treated with 20 μg/mL rFXIIIa or medium containing inhibited thrombin for 24 h after cell seeding. After 24 h, cells were washed with PBS and fixed by 3% glacial acetic acid in 96% ethyl alcohol for 10 min. After washing with PBS, the surface was blocked with 5% normal human serum for 15 min. Biotinylated mouse monoclonal anti-TSP-1 (ThermoFisher) (1:200 dilution, 60 min) and horse anti-mouse IgG DyLight 488 (Vector Laboratories) (1:100 dilution, 45 min) were used for TSP-1 immunofluorescent staining. The slides were covered by Vectashield Antifade Mounting Medium with DAPI (Vector Laboratories). Slides were investigated with AxioImage. M2 fluorescence microscope (Zeiss Oberkochen, Germany) and representative images were acquired by confocal laser scanning microscope (LSM 700, Zeiss Oberkochen, Germany) equipped with Plan-Apochromat 63 x/1.40 oil objective and solid-state diode lasers (405 nm, 488 nm and 555 nm). Detection of the fluorescence signals was performed by selective laser excitation coupled to efficient splitting of the emitted light using variable secondary dichroic (VSD) beam-splitter.

### 4.11. Real-Time PCR

Cells were seeded into 24-well plates at a density of 3 × 10^4^ cells/well. After 24 h, they were treated with different concentrations of rFXIIIa or non-activated rFXIII-A_2_. In certain experiments, treatments were repeated daily for 3 days. QIAamp RNA Blood Mini kit (Qiagen, Hilden, Germany) was used for the extraction of total RNA from the cells. The extracted RNA was reverse-transcribed into cDNA by qPCRBIO cDNA Synthesis Kit (Nucleotest Bio Ltd., Budapest, Hungary). cDNA samples were amplified using LightCycler^®^480 SYBR Green I Master Mix (Roche, Basel, Switzerland). The specific primers used for TSP-1 and GAPDH mRNA detection were described by Dardik et al. [27,33].

### 4.12. Statistical Analysis

Student’s *t*-test was used to assess statistical significance, and *p* < 0.05 was considered statistically significant. Means represent the results of at least 6 individual measurements.

## Supplementary Materials

The following supporting information can be downloaded at: https://www.mdpi.com/article/10.3390/ijms23105845/s1.

## Abbreviations

CM	Calcification medium
cFXIII	Cellular factor XIII
FXIIIa	Activated factor XIII
FXIII	Blood coagulation factor XIII
FXIII-A	Factor XIII A subunit
FXIII-B	Factor XIII B subunit
GAPDH	Glyceraldehyde 3-phosphate dehydrogenase
GM	Growth medium
HAoSMC	Human aortic smooth muscle cell
pFXIII	Plasma factor XIII
rFXIII	Recombinant factor XIII
TSP-1	Thrombospondin-1
VSMC	Vascular smooth muscle cell
