# Supplementary material for: The Effect of Activated FXIII, a Transglutaminase, on Vascular Smooth Muscle Cells
Source: Int J Mol Sci. 2022 May 23;23(10):5845. doi: 10.3390/ijms23105845 (PMC9144255; doi:10.3390/ijms23105845)
Supplement: Supplementary file 1 [file ijms-23-05845-s001.zip › ijms-1704036-supplementary/Supplementary materials/Supplementary Material.pdf]

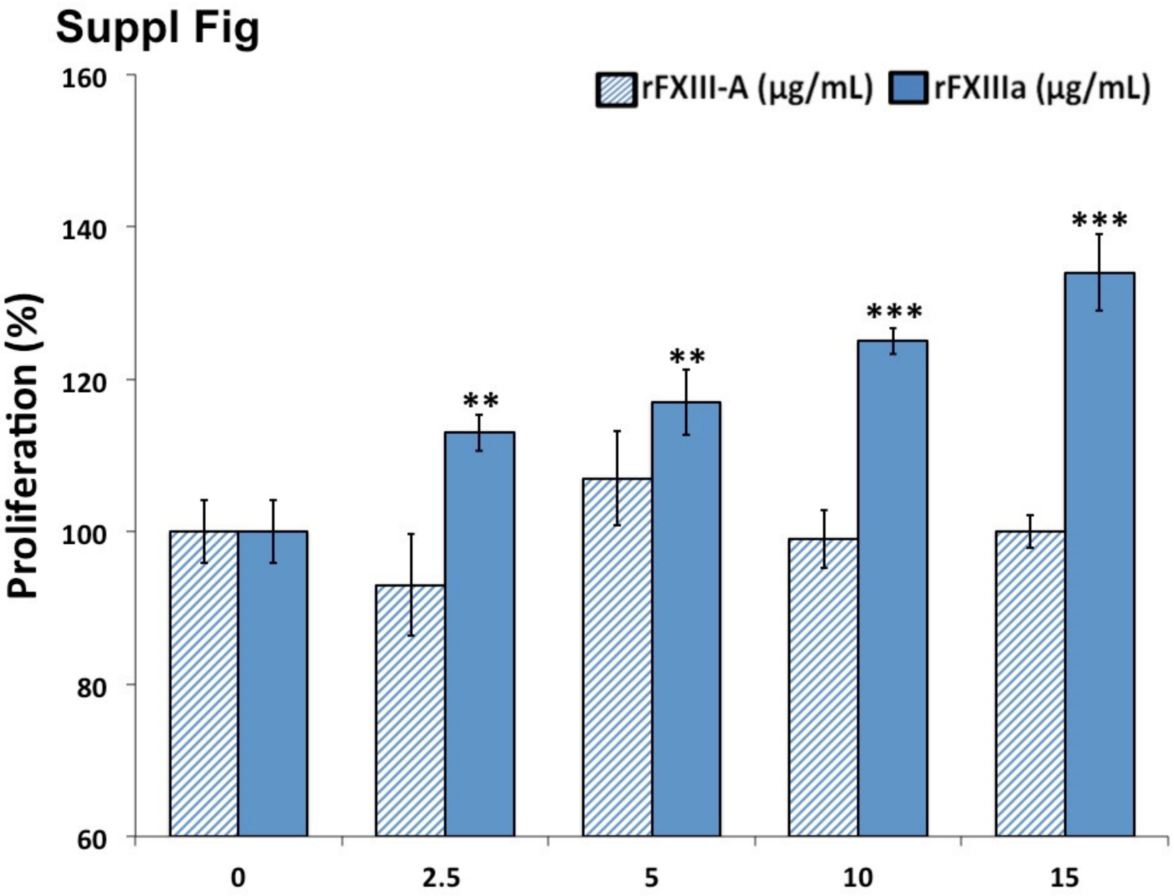

Supplementary Figure S1. rFXIIIa enhances the proliferation of human aortic smooth muscle cells (HAoSMCs) as measured by the CCK-8 cell proliferation assay. The proliferation of rFXIIIa treated cells is expressed as percentage of the proliferation by non-treated cells. As demonstrated by shaded columns non-activated rFXIII-A_2 failed to influence the proliferation of HAoSMCs. The assay monitors the reduction of tetrazolium salt to colored formazan at 450 nm. ***P*<0.01, ****P*<0.001

Supplementary Video S1. Monitoring the closure of in vitro gap wound in human aortic smooth muscle cell culture in the absence and in the presence of 20 μg/mL activated recombinant FXIII (rFXIIIa). After removing the insert from the cell culture, the gradual closure of the cell free gap by proliferating and migrating cells was monitored by real time microscopy.
